# *In Vitro* Activity of Chlorhexidine Compared with Seven Antifungal Agents against 98 *Fusarium* Isolates Recovered from Fungal Keratitis Patients

**DOI:** 10.1128/AAC.02669-18

**Published:** 2019-07-25

**Authors:** Claudy Oliveira dos Santos, Eva Kolwijck, Henrich A. van der Lee, Marlou C. Tehupeiory-Kooreman, Abdullah M. S. Al-Hatmi, Einoti Matayan, Matthew J. Burton, Cathrien A. Eggink, Paul E. Verweij

**Affiliations:** a Department of Medical Microbiology, Radboud University Medical Center, Nijmegen, The Netherlands; b Center of Expertise in Mycology Radboudumc/CWZ, Nijmegen, The Netherlands; c Department of Medical Microbiology, University of Groningen, University Medical Center Groningen, Groningen, The Netherlands; d Westerdijk Fungal Biodiversity Institute, Utrecht, The Netherlands; e Directorate General of Health Services, Ministry of Health, Ibri Hospital, Ibri, Oman; f Department of Ophthalmology, Kilimanjaro Christian Medical Center, Moshi, Tanzania; g International Center for Eye Health, London School of Hygiene and Tropical Medicine, London, United Kingdom; h Department of Ophthalmology, Radboud University Medical Center, Nijmegen, The Netherlands

**Keywords:** *Fusarium*, antifungal susceptibility testing, keratitis, microbiology, mycology

## Abstract

Fungal keratitis is a common but severe eye infection in tropical and subtropical areas of the world. In regions with a temperate climate, the frequency of infection is rising in patients with contact lenses and following trauma. Early and adequate therapy is important to prevent disease progression and loss of vision. The management of *Fusarium* keratitis is complex, and the optimal treatment is not well defined. We investigated the *in vitro* activity of chlorhexidine and seven antifungal agents against a well-characterized collection of *Fusarium* isolates recovered from patients with *Fusarium* keratitis. The fungus culture collection of the Center of Expertise in Mycology Radboudumc/CWZ was searched for *Fusarium* isolates that were cultured from cornea scrapings, ocular biopsy specimens, eye swabs, and contact lens fluid containers from patients with suspected keratitis. The *Fusarium* isolates that were cultured from patients with confirmed keratitis were all identified using conventional and molecular techniques. Antifungal susceptibility testing was performed according to the EUCAST broth microdilution reference method. The antifungal agents tested included amphotericin B, voriconazole, posaconazole, miconazole, natamycin, 5-fluorocytosine, and caspofungin. In addition, the activity of chlorhexidine was determined. The fungal culture collection contained 98 *Fusarium* isolates of confirmed fungal keratitis cases from 83 Dutch patients and 15 Tanzanian patients. The isolates were collected between 2007 and 2017. Fusarium oxysporum (*n* = 24, 24.5%) was the most frequently isolated species followed by Fusarium solani *sensu stricto* (*n* = 18, 18.4%) and Fusarium petroliphilum (*n* = 11, 11.2%). Amphotericin B showed the most favorable *in vitro* inhibition of *Fusarium* species followed by natamycin, voriconazole, and chlorhexidine, while 5-fluorocytosine, posaconazole, miconazole, and caspofungin showed no relevant inhibiting effect. However, chlorhexidine showed fungicidal activity against 90% of F. oxysporum strains and 100% of the F. solani strains. Our study supports the clinical efficacy of chlorhexidine and therefore warrants its further clinical evaluation for primary therapy of fungal keratitis, particularly in low and middle income countries where fungal keratitis is much more frequent and, currently, antifungal eye drops are often unavailable.

## Abstract (precis)

Fungal keratitis is a common but severe eye infection in tropical and subtropical areas of the world. In regions with a temperate climate, the frequency of infection is rising in patients with contact lenses and following trauma. Early and adequate therapy is important to prevent disease progression and loss of vision. The management of *Fusarium* keratitis is complex, and the optimal treatment is not well defined.

## INTRODUCTION

Fungal keratitis is a common eye infection in tropical and subtropical areas of the world. In regions with a temperate climate, fungal keratitis is uncommon but mainly reported in patients with contact lenses and following trauma. Early therapy is important to prevent disease progression or dissemination. A major complication of fungal keratitis is monocular blindness, especially in tropical low and middle income countries (LMIC), where significant delay in diagnosis and simply the unavailability of antifungals are common. Most cases of fungal keratitis are caused by *Fusarium* species (1, 2), which are ubiquitous fast-growing hyalohyphomycetes that are present in soil, water, and plants. The most common route of infection is by (micro)trauma or disruptive ocular surface disease, as filamentous fungi are unable to penetrate intact cornea.

The taxonomy of the *Fusarium* order is complex and still not well defined. Molecular techniques have shown that the common medically relevant species, Fusarium solani and Fusarium oxysporum, consist of multiple (sub)species (3). Although *in vitro* antifungal susceptibility patterns of *Fusarium* species may vary greatly within each species, most species show high MICs to the currently licensed antifungals (4–7). The European Committee on Antimicrobial Susceptibility Testing (EUCAST) has not defined epidemiological cutoff (ECOFF) values and clinical breakpoints for *Fusarium* species. In 2015, Espinel-Ingroff et al. published epidemiological cutoff values (ECVs) based on the Clinical and Laboratory Standards Institute (CLSI) broth dilution method for antifungal susceptibility testing (AFST) (8). As topical antifungal therapy is important for fungal keratitis management, a meaningful classification of isolates as resistant or susceptible is challenging. These factors complicate the management of *Fusarium* keratitis (9), and the optimal treatment is not well defined.

Currently, a 0.02% chlorhexidine solution is used for the treatment of *Acanthamoeba* keratitis. A few studies have indicated that the disinfectant chlorhexidine might be an effective, affordable, and accessible treatment for fungal keratitis, which could benefit millions of people who currently have no options (10, 11). The meta-analysis in the Cochrane systematic review written by FlorCruz and Evans show that chlorhexidine has a better clinical outcome than natamycin and voriconazole (12). To our knowledge, there are no published data describing the MICs of chlorhexidine for *Fusarium* species. In this study, we investigated the *in vitro* activity of chlorhexidine and seven antifungal agents against a molecularly characterized set of *Fusarium* isolates recovered from patients with keratitis.

## RESULTS

The fungal culture collection contained 98 *Fusarium* isolates from 83 patients with keratitis from the Netherlands and 15 with keratitis from Tanzania. The isolates were collected between 2007 and 2017. The first *Fusarium* isolate per patient was tested.

Molecular identification showed that F. oxysporum (*n* = 24, 24.5%) was the most frequently isolated species followed by F. solani *sensu stricto* (*n* = 18, 18.4%) and Fusarium petroliphilum (*n* = 11, 11.2%). Based on the assignment of the isolates to the according species complex, as described by Salah et al. (3), the most frequently encountered complexes were F. solani species complex (FSSC; *n* = 43, 43.9%), F. oxysporum species complex (FOSC; *n* = 24, 24.5%), Fusarium fujikuroi species complex (FFSC; *n* = 16, 16.3%) and Fusarium dimerum species complex (FDSC; *n* = 12, 12.2%). One isolate could not be assigned to any species complex or species and appears to be a new *Fusarium* species.

The MIC distributions for the various species and species complexes are shown in Tables 1 and 2. *In vitro* amphotericin B was the most active antifungal drug followed by natamycin, voriconazole, posaconazole, and miconazole. Chlorhexidine showed activity against all species at a concentration of 8 to 32 mg/liter, which corresponds with 1.56 × 10^−3^% to 6.25 × 10^−3^%. 5-Fluorocytosine showed no *in vitro* activity.

**TABLE 1:** Molecularly identified *Fusarium* keratitis isolates and their susceptibility profiles to eight antifungal agents, including chlorhexidine and natamycin

*Fusarium* species (*n*)	MIC (%) (median [range])	MIC (mg/liter) (median [range])^*a*^							MEC^*b*^ mg/liter (median [range])
	CHX	CHX	AMB	VCZ	5-FC	MCZ	NAT	POS	CAS^*c*^
*Fusarium* species (1)	0.003	16	0.5	2	32	16	8	16	16
F. falciforme (7)	0.006 (0.002–0.006)	32 (8–32)	2 (1–8)	16 (8–16)	>32 (>32)	16 (16)	8 (8–16)	16 (16)	16 (16)
F. keratoplasticum (7)	0.003 (0.002–0.006)	16 (8–32)	4 (2–4)	8 (4–16)	>32 (>32)	16 (16)	4 (4–8)	16 (16)	16 (0.5–16)
F. petroliphilum (11)	0.003 (0.002–0.006)	16 (8–32)	2 (0.5–4)	8 (4–16)	>32 (>32)	16 (16)	4 (4–8)	16 (16)	16 (2–16)
F. solani (18)	0.006 (0.002–0.006)	32 (8–32)	2 (0.063–16)	8 (4–16)	>32 (>32)	16 (8–16)	8 (4–16)	16 (8–16)	16 (4–32)
F. oxysporum (24)	0.002 (0.0002–0.012)	8 (1–64)	2 (0.25–16)	4 (2–16)	32 (0.063–32)	16 (16)	8 (4–8)	16 (16)	16 (0.063–32)
F. musae (1)	0.003	16	2	4	>32	8	8	1	16
F. verticillioides (3)	0.003 (0.001–0.003)	16 (4–16)	2 (1–8)	2 (1–2)	>32 (>32)	1 (0.25–8)	8 (2–8)	0.5 (0.25–1)	16 (16)
F. proliferatum (7)	0.002 (0.001–0.012)	8 (4–64)	2 (1–4)	4 (2–8)	>32 (>32)	16 (16)	8 (4–8)	4 (2–16)	16 (16)
F. ramigenum (1)	0.003	16	4	1	>32	16	4	1	16
F. sacchari (1)	0.002	8	2	1	>32	4	8	0.25	16
F. lactis (3)	0.002 (0.002–0.003)	8 (8–16)	2 (0.5–4)	4 (2–8)	>32 (>32)	16 (16)	8 (8)	16 (2–16)	16 (16)
F. equiseti (1)	NP^*d*^	NP	1	8	NP	NP	NP	16	32
F. dimerum (8)	0.002 (0.002–0.003)	8 (8–16)	1 (0.5–2)	8 (4–8)	>32 (>32)	16 (16)	4 (4–16)	16 (16)	16 (2–16)
F. delphinoides (4)	0.001 (0.001–0.002)	4 (4–8)	0.5 (0.125–1)	2 (2)	>32 (>32)	16 (16)	4 (2–4)	8 (1–16)	16 (2–16)
F. ambrosium (1)	0.006	32	2	16	>32	16	8	16	1

a CHX, chlorhexidine; AMB, amphotericin B; VCZ, voriconazole; 5FC, 5-fluorocytosine; MCZ, miconazole; NAT, natamycin; POS, posaconazole.

b MEC, minimal effective concentration.

c CAS, caspofungin.

d NP, susceptibility testing for this antifungal agent was not performed.

**TABLE 2:** *Fusarium* keratitis isolates assigned according to the species complex and their susceptibility profile to eight antifungal agents, including chlorhexidine and natamycin

*Fusarium* species complex (*n*)^*a*^	MIC (%) (median [range])	MIC (mg/liter) (median [range])^*b*^							MEC^*c*^ mg/liter (median [range])
	CHX	CHX	AMB	VCZ	5-FC	MCZ	NAT	POS	CAS^*d*^
Unknown (1)	0.003	16	0.5	2	32	16	8	16	16
FSSC (43)	0.003 (0.002–0.006)^*e*^	16 (8–32)	2 (0.063–16)	8 (4–16)^*e*^	>32 (>32)	16 (8–16)	8 (4–16)	16 (8–16)	16 (0.5–32)
FOSC (24)	0.002 (0.002–0.012)	8 (2–64)	2 (0.25–16)	4 (2–16)	32 (0.063–32)	16 (16)	8 (4–8)	16 (16)	16 (0.063–32)
FFSC (16)	0.002 (0.001–0.012)	8 (4–64)	2 (0.5–8)	4 (1–8)	>32 (>32)	16 (0.25–16)^*e*^	8 (2–8)	2 (0.25–16)^*e*^	16 (16)
FIESC (1)	NP^*f*^	NP	1	8	NP	NP	NP	16	32
FDSC (12)	0.002 (0.0008–0.003)	8 (4–16)	1 (0.125–2)^*e*^	8 (2–8)	>32 (>32)	16 (16)	4 (2–16)^*e*^	16 (1–16)	16 (2–16)
AFC (1)	0.006	32	2	16	>32	16	8	16	1

a FSSC, F. solani species complex; FOSC, F. oxysporum species complex; FFSC, *F. fujikuroi* species complex; FIESC, F. incarnatum-*equiseti* species complex; FDSC, F. dimerum species complex; AFC, Ambrosia *Fusarium* clade.

b CHX, chlorhexidine; AMB, amphotericin B; VCZ, voriconazole; 5FC, 5-fluorocytosine; MCZ, miconazole; NAT, natamycin; POS, posaconazole.

c MEC, minimal effective concentration.

d CAS, caspofungin.

e Significant difference of the median and or distribution range between the groups of species complex.

f NP, susceptibility testing for this antifungal agent was not performed.

### Statistics.

The median MICs and the MIC distributions of 5-fluorocytosine and caspofungin showed no differences between any of the groups.

The MIC distributions of amphotericin B showed significant differences between the species complexes (Kruskal Wallis test, *P* = 0.002). FDSC differed significantly from FSSC and from FFSC.

For voriconazole, the median MICs and the MIC distributions showed significant differences between the species complexes (*P* = 0.006 and *P* = 0.000, respectively, Kruskal Wallis test). FSSC differed significantly from FOSC, from FFSC, and from FDSC.

The MIC distributions of posaconazole and miconazole showed significant differences between the species complexes (*P* = 0.000 and *P* = 0.001, respectively, Kruskal Wallis test). For posaconazole and miconazole, FFSC differed significantly from FSSC and from FOSC.

The median MICs of natamycin were not different between the species complex (SC) groups (*P* = 0.747, Kruskal-Wallis). On the other hand, the MIC distributions of natamycin differed significantly between the species complexes (*P* = 0.015, Kruskal Wallis test). FDSC differed significantly from FSSC, from FOSC, and from FFSC.

The median MICs and the MIC distributions of chlorhexidine showed significant differences between the species complexes (*P* = 0.000 and *P* = 0.000, respectively, Kruskal Wallis test). FSSC differed significantly from FOSC, from FFSC, and from FDSC.

MIC values of posaconazole, miconazole, 5-fluorocytosine, and caspofungin were high, and so determining the minimal fungicidal concentration (MFC) of these agents was deemed clinically not relevant.

**FIG 1:**
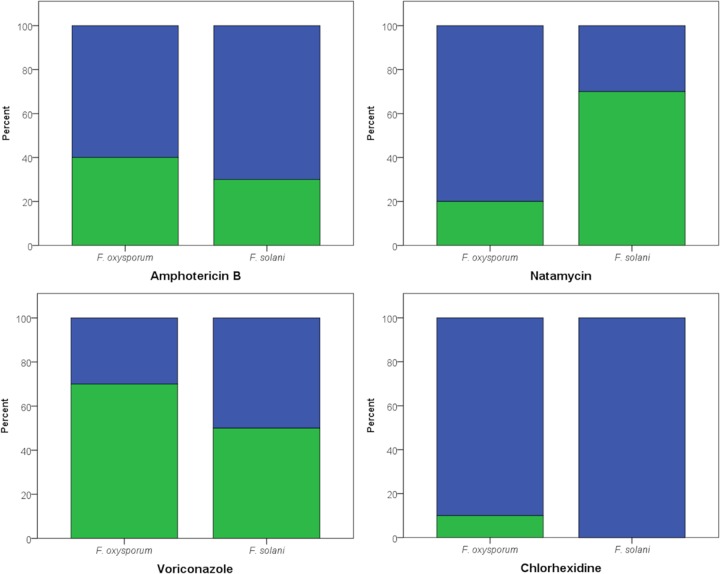
The proportions of fungicidal (blue) and fungistatic (green) *in vitro* effects of amphotericin B, natamycin, voriconazole, and the disinfectant chlorhexidine depicted for Fusarium oxysporum (*n* = 10) and Fusarium solani (*n* = 10), all of which were isolated from patients with fungal keratitis.

*In vitro* amphotericin B exhibited fungicidal effect on 60% of the F. oxysporum strains and 70% of the F. solani strains; the remainder of the strains showed a fungistatic effect (Fig. 1). Natamycin was fungicidal against 80% of the F. oxysporum strains. However, in F. solani strains, natamycin was mostly fungistatic; in only 30%, it acted as a fungicidal. Voriconazole was fungicidal against 30% of the F. oxysporum strains and 50% of the F. solani strains. Chlorhexidine showed fungicidal activity against 90% of F. oxysporum strains and 100% of the F. solani strains.

## DISCUSSION

Chlorhexidine showed broad *in vitro* activity against all *Fusarium* species tested and, compared with the antifungal agents, showed the broadest fungicidal activity against the two species tested. Although it is likely that chlorhexidine is fungicidal in other *Fusarium* species, this was not tested. For chlorhexidine, 95% of the 20 *Fusarium* isolates were killed at concentrations far below the 0.02% and 0.2% concentrations, of which, the 0.02% concentration for eye drops is already commonly used by ophthalmologists for treatment of *Acanthamoeba* keratitis. Another important advantage of chlorhexidine gluconate solution is the broad antimicrobial spectrum, including against Gram-positive and Gram-negative bacteria, lipid-enveloped viruses, and *Acanthamoeba* (12, 13).

A limited number of clinical trials have studied the effectiveness of chlorhexidine gluconate for the treatment of fungal keratitis. The aim of one trial was to find the most effective dose of chlorhexidine (14). In comparison to the response with natamycin, the relative efficacy in a patient without prior antifungal treatment was 1.17 with chlorhexidine 0.05%, 1.43 with chlorhexidine 0.1%, and 2.00 with chlorhexidine 0.2%. Their fungal isolates were not subjected to molecular identification and susceptibility testing. The second study by Rahman et al. (10) showed a relative efficacy of 1.85 (confidence interval [CI], 1.01 to 3.39; *P* = 0.04) with chlorhexidine 0.2% in comparison to natamycin. Of the nonsevere ulcers, 66.7% were healed at day 21 with chlorhexidine and 36.0% with natamycin. However, this trial was not double blinded due to the fact that personnel could identify the selected treatment because the solutions of chlorhexidine and natamycin were visibly different (10). The susceptibility testing was performed with a nonreference well diffusion method (0.2% chlorhexidine, 2.5% natamycin, 1% econazole). The concentration of natamycin used was 2.5%, which is half the current standard therapeutic concentration of 5%. In addition, the method of identification of the strains was also not mentioned.

In the Netherlands, the available antifungal agents which can be used as eye drops are amphotericin B 0.15% (1,500 mg/liter), voriconazole 1% (10,000 mg/liter), and the disinfectant chlorhexidine 0.02% (200 mg/liter). These formulas are not commercially available but are prepared by hospital pharmacists on request. In other countries, natamycin (5% suspension; 50,000 mg/liter) is available and frequently used in the setting of fungal keratitis (2, 10–12, 15–20). These concentrations exceed by far the *in vitro* determined MICs of the *Fusarium* isolates (Tables 1 and 2). However, effectiveness depends on many factors, including the ability of the compound to penetrate ocular tissues, local bioavailability, and drug toxicity.

The most important route of penetration of topical antifungals into ocular tissue is through the cornea, mostly by diffusion. The polyenes, amphotericin B and natamycin, are compounds with a high molecular mass (i.e., >500 Da) and, as a consequence, barely penetrate intact cornea epithelium (21). This leads to the necessity of regularly performing abrasions of the cornea during treatment with amphotericin B. In high doses, amphotericin B can be toxic to the cornea, but the 0.15% solution is well tolerated (21). Due to the viscous nature of natamycin suspension and its poor penetration, it is only suitable for treatment of superficially located keratomycosis. Furthermore, compounds that are lipophilic can penetrate across the corneal stroma, while hydrophilic agents are able to penetrate all the layers of the cornea.

There is little known about the corneal penetration of the cationic antisepticum chlorhexidine gluconate. In a small animal study, Vontobel et al. showed that the compound did not penetrate through the intact or mechanically damaged cornea into the anterior chamber (22). It appears that chlorhexidine accumulates within the cornea, explaining the need to treat deep-seated *Acanthamoeba* for a long time.

Amphotericin B showed the most favorable *in vitro* inhibition of *Fusarium* species, followed by natamycin, voriconazole, and chlorhexidine, while 5-fluorocytosine, posaconazole, miconazole, and caspofungin showed no relevant inhibiting effect. However, chlorhexidine showed fungicidal activity against 90% of F. oxysporum strains and 100% of the F. solani strains.

The differences in AFST between isolates belonging to the same species complex justifies conducting molecular identification to the species complex level. In general, the species belonging to the FDSC and the FFSC are more susceptible to chlorhexidine, amphotericin B, natamycin, voriconazole, and posaconazole (only FFSC). These differences cannot be predicted by identification based on conventional methods, because the characteristics of morphology and microscopy are not species specific.

Our study supports the clinical efficacy of chlorhexidine and therefore warrants its further clinical evaluation for primary therapy of fungal keratitis, particularly in LMIC where fungal keratitis is much more frequent and, currently, antifungal eye drops are often unavailable. Further studies should investigate the *in vitro* interaction of chlorhexidine with antifungal agents to support alternate administration and combination therapy.

## MATERIALS AND METHODS

The fungus culture collection of the Center of Expertise in Mycology Radboudumc/CWZ was searched for *Fusarium* isolates that were cultured from cornea scrapings, ocular biopsy specimens, eye swabs, and contact lens fluid containers from patients with suspected keratitis. All isolates had been identified to the genus level using conventional techniques. For accurate species identification, sequencing of the *TEF1* gene was performed (3).

Antifungal susceptibility testing was performed according to the EUCAST broth microdilution reference method (23, 24). The antifungal agents tested included amphotericin B (Bristol Myers Squibb), voriconazole (Pfizer), posaconazole (Merck & Co), miconazole (Janssen Cilag), natamycin (Sigma-Aldrich), 5-fluorocytosine (Hoffman la Roche), and caspofungin (Merck & Co). In addition, the activity of chlorhexidine (Pharmaline) was determined. The test range of the antifungal agents was 0.02 to 16 mg/liter for amphotericin B, voriconazole, posaconazole, miconazole, caspofungin, and natamycin, and 0.03 to 32 mg/liter for 5-fluorocytosine. For chlorhexidine, a concentration range of 1 to 1,024 mg/liter was used, which corresponds to a range of 0.000195% to 0.2%. All antifungal agents and chlorhexidine were dissolved in RPMI 1640 supplemented with glucose to a final concentration of 2%. The MICs were determined with an inverted mirror after 48 h at 35°C as the lowest drug concentration with complete inhibition of growth visible by eye for amphotericin B, voriconazole, posaconazole, miconazole, natamycin, 5-fluorocytosine, and chlorhexidine. The endpoint for echinocandins was the minimal effective concentration (MEC). The MEC for caspofungin was determined with an inverted microscope after 48 h at 35°C as the lowest drug concentration in which abnormal, short, and branched hyphal clusters were observed in contrast to the long, unbranched, elegant hyphal elements that were visible in the growth control well. Aspergillus fumigatus ATCC 204305 and Aspergillus flavus ATCC 204304 were used as quality control strains as recommended by the EUCAST (23).

Ten F. oxysporum and 10 F. solani isolates were used to determine the minimal fungicidal concentration (MFC) for the antifungal agents and chlorhexidine. After reading the MICs at 48 h, the 96-wells plates were shaken to loosen the fungal elements. Thereafter, 20 μl from all the wells with no visible growth and 20 μl from the growth control were plated on Sabouraud agar (Oxoid). The plates were incubated for 24 and 48 h at 35°C. The MFC was determined as the lowest drug concentration which led to 99% to 99.5% growth inhibition compared to the growth control. Antifungal agents were considered fungicidal when the MIC value was no more than two dilution steps lower than the MFC (25). If the difference between MIC and MFC was >2 dilution steps, the antifungal agent was classified as fungistatic (25).

Statistical analysis was performed with IBM SPSS Statistics 24. *In vitro* susceptibility differences between *Fusarium* species and differences between species complexes were tested with a nonparametric test (one-way analysis of variance [ANOVA], Kruskal-Wallis). A *P* value of <0.05 was determined as significant. To correct for multitesting in the search for which groups differed from each other, the *P* value was adjusted according to Bonferroni’s correction method (e.g., significance level [<0.05] divided by the number of tests needed). Bonferroni’s correction for the species groups was a *P* value of <0.0011; for the species complex groups, Bonferroni’s *P* value was <0.0083. Species and species complexes with only one isolate where not taken into account in the statistical analysis. For every antifungal or antiseptic agent, we tested for significant differences between the species complexes by comparing the median MICs or percentages of the concentration and comparing the distributions between the groups. After this comparison, the groups which were responsible for the significant difference were determined by comparing one group to another group with the Mann-Whitney U test.

The samples from Dutch participants were collected during the routine standard of care. Therefore, we did not need their informed consent in accordance with the Dutch Ethics Committee of the Radboud University Medical Center.

The collection of samples from Tanzanian participants was approved by the Ethics Committees of the National Institute for Medical Research, Tanzania, and the London School of Hygiene & Tropical Medicine, United Kingdom. Informed consent was obtained from all participants.
